# Modifying *Escherichia coli* to mimic *Shigella* for medical microbiology laboratory teaching: a new strategy to improve biosafety in class

**DOI:** 10.3389/fcimb.2023.1257361

**Published:** 2023-09-13

**Authors:** Guangyuan Zhang, Jia Liu, Yonglin He, Yuheng Du, Lei Xu, Tingting Chen, Yanan Guo, Huichao Fu, Anlong Li, Yunbo Tian, Yan Hu, Chun Yang, Mingqi Lu, Xichuan Deng, Jingsong Wang, Nan Lu

**Affiliations:** ^1^ Chongqing Medical University, Basic Medical School, Department of Pathogen Biology, Chongqing, China; ^2^ Chongqing Medical University, Pathogen Biology and Immunology Laboratory and Laboratory of Tissue and Cell Biology, Experimental Teaching and Management Center, Chongqing, China; ^3^ Department for Rehabilitation Medicine, The Second College of Clinical Medicine of Chongqing Medical University, Chongqing, China; ^4^ Chongqing Medical University, International Medical College, Chongqing, China; ^5^ Chongqing Blood Center, Quality Management Section, Chongqing, China; ^6^ Tuberculosis Reference Laboratory, Chongqing Tuberculosis Control Institute, Chongqing, China; ^7^ R&D Department, Chongqing Kebilong Biotechnology Co., Ltd., Chongqing, China

**Keywords:** medical microbiology laboratory class teaching, biosafety, *Escherichia coli* DH5α, *Shigella*, substitution materials

## Abstract

**Introduction:**

Laboratory teaching of medical microbiology involves highly pathogenic microorganisms, thus posing potential biosafety risks to the students and the teacher. To address these risks, non/low-pathogenic microorganisms were modified to mimic highly pathogenic ones or highly pathogenic microorganisms were attenuated directly using the CRISPR/Cas9 technology. This study describes the modification of *Escherichia coli* DH5α to mimic *Shigella* and its evaluation as a safe alternative for medical laboratory teaching.

**Methods:**

To generate *E. coli* DH5α△FliC△tnaA2a, the tnaA and FliC genes in *E. coli* DH5α were knocked out using CRISPR/Cas9 technology; a plasmid bearing the O-antigen determinant of *S. flexneri* 2a was then constructed and transformed. Acid tolerance assays and guinea pig eye tests were used to assess the viability and pathogenicity, respectively. Questionnaires were used to analyze teaching effectiveness and the opinions of teachers and students.

**Results:**

The survey revealed that most teachers and students were inclined towards real-time laboratory classes than virtual classes or observation of plastic specimens. However, many students did not abide by the safety regulations, and most encountered potential biosafety hazards in the laboratory. *E. coli* DH5α△FliC△tnaA2a was biochemically and antigenically analogous to *S. flexneri* 2a and had lower resistance to acid than *E. coli*. There was no toxicity observed in guinea pigs. Most of teachers and students were unable to distinguish *E. coli* DH5α△FliC△tnaA2a from pure *S. flexneri* 2a in class. Students who used *E. coli* DH5α△FliC△tnaA2a in their practice had similar performance in simulated examinations compared to students who used real *S. flexneri* 2a, but significantly higher than the virtual experimental group.

**Discussion:**

This approach can be applied to other high-risk pathogenic microorganisms to reduce the potential biosafety risks in medical laboratory-based teaching and provide a new strategy for the development of experimental materials.

## Introduction

Medical microbiology is an important part of basic medicine, and has become highly significant with the advent of COVID-19 and other bacterial diseases (Lee et al., 2020; Ikuta et al., 2022). Practical laboratory course is an indispensable and the most attractive part of medical microbiology. However, medical microbiology laboratory courses involve live pathogenic microorganisms, many of which are highly pathogenic and require operation in at least biosafety level II laboratories (Mareedu-Boada et al., 2021). Consequently, undergraduate laboratory classes commonly adopt measures such as safety education, strict supervision, constructing high biosafety level laboratories and strict implementation of safety protocols, including counting inocula after class, stringent sterilization, etc (Vijayan et al., 2019; Townsend and Goffe, 2022). Other measures, such as virtual simulation experiments were also used to maximize students’ proficiency and minimize biosafety risks (Makransky et al., 2016; Cardoso et al., 2021).

Despite these measures, laboratory-acquired infections and even deaths are reported every year (Wurtz et al., 2016). Twenty-eight students from the Northeast Agricultural University in China were infected with Brucella during a laboratory class, similarly Lanzhou Institute of Biological Products also faced an outbreak of bacterial infection (Song et al., 2021; Liu et al., 2022). Indicating that these measures alone are insufficient in completely avoiding risks, let alone many schools lack the infrastructure to provide enough biological security such as high-level biosafety laboratories, virtual equipment or appropriate student-teacher ratio in the laboratory course. These result in potential biosafety mishaps due to inadequate supervision or incorrect operation. Therefore, relevant experimental content had to be removed or replaced with low-quality substitutes such as playing videos or teacher demonstration which hindered students’ understanding of related pathogenic microorganisms.

Consequently, newer approaches are essential for increasing biosafety while maintaining or improving the teaching quality in a medical laboratory class. This study entails modifying non-/low pathogenic microorganisms to mimic highly pathogenic microorganisms or vice versa, by attenuating highly pathogenic microorganisms using CRISPR/Cas9 technology, for practical training (Jiang et al., 2015; Yan et al., 2017; Hashemi, 2020). Specifically, we knocked out tnaA and FliC genes in *E. coli* DH5α using CRISPR/Cas9 to generate a surrogate for *Shigella*, *E. coli* DH5α△FliC△tnaA2a. A plasmid bearing the O-antigen determinant of *S. flexneri* 2a was then constructed and transformed. We then evaluated the safety and resistance of the surrogate *E. coli* DH5α△FliC△tnaA2a and employed it in undergraduate laboratory teaching. Feedback from both teachers and students demonstrated the feasibility of this approach, indicated that they will facilitate the teaching and improve the biosafety of medical microbiology laboratory courses.

## Materials and methods

### Bacteria, culture media, plasmid and pathway analysis

Luria-Bertani (LB), Roche, Kligler Iron Agar (KIA), MacConkey Agar Media (MAC), Motility Indol Urea Media Agar (MIU), and *Salmonella* and *Shigella* (SS) Agar media were prepared in our lab. The Gram stain and acid-fast staining kit were purchased from Sigma (USA). Taq and PFU DNA polymerase, as well as all restriction endonucleases, were purchased from NEB (USA). Primers were synthesized by Tsingke Biotechnology Co. (China) and the information were listed in **Table S1**. *E. coli* DH5α was purchased from Takara (Japan). *S. flexneri* 2a, *E. coli* (ATCC 25922), *Staphylococcus saprophyticus* (ATCC 15305), *S. aureus* (ATCC 8095), *Mycobacterium smegmatis* (ATCC 14468), and *M. tuberculosis* H37Rv (ATCC 27294) were obtained from the experimental teaching center of Chongqing Medical University. The diagnostic sera kit for *Shigella* was purchased from Ningbo Tianrun Bio-pharmaceutical Co., LTD (China). The pOcus2 plasmid and simulated plastic specimens were kept in our lab and described previously (Jia et al., 2022; Zhang et al., 2013). The key enzymes of tryptophan metabolism pathway and the genes encoding flagella of *E. coli* were analyzed on Kyoto Encyclopedia of Genes and Genomes (https://www.kegg.jp/).

### Clone and expression of *S. flexneri* 2a O antigen determinant in *E. coli* DH5α△TnaA△FliC

The generation of tnaA and FliC gene deficient *E. coli* DH5α△TnaA△FliC were described in **supplementary data**. On this base, a plasmid pOcus2-Sf2aO bearing the O-antigen determinant of *S. flexneri* 2a was constructed and transformed into the *E. coli* DH5α△TnaA△FliC to generate *E. coli* DH5α△TnaA△FliC2a which consistently express the *S. flexneri* 2a O antigen. The expression of *S. flexneri* 2a O antigen was confirmed using a serological agglutination test following the protocol of the kit and the plasmid pOcus2-Sf2aO were constructed in the following steps.

First, the backbone of the pOcus2 plasmid, including the replication origin sites and the ampicillin-resistant gene, was amplified using the primers T7-ter-F(ln) and pOcus2-R(ln). Then, a 2443bp fragment containing the bgt and grtII genes, and a 3984bp fragment containing the rfbB-C gene were amplified separately from the genome of *S. flexneri* 2a using the primer pairs bgt-F(ln)/grtII-R(ln) and rfbB-F/rfbC-R, respectively.Thereafter, all three fragments were ligated together using the Seamless Cloning Kit (Biorun, China) and transformed into normal *E. coli* DH5α competent cells to generate the intermediate plasmid pOcus-bgtII-rfbC. Finally, a 6233bp fragment was amplified from the genome of *S. flexneri* 2a using the primers frbC-F and prMD4-2A and inserted into the XhoI site of the intermediate plasmid pOcus-bgtII-rfbC using the Seamless Cloning Kit to generate the *S. flexneri* 2a O antigen expression plasmid, pOcus2-Sf2aO.

### Guinea pig eyes challenge assay and acid tolerance assay

Female guinea pigs, three weeks old, were purchased from the Laboratory Animal Center at Chongqing Medical University and housed in cages that comply with animal welfare standards. After anesthesia, both eyes of each guinea pig (n = 4 per group) were inoculated with 5x10^6^/20 μL bacteria in PBS. The severity of eye inflammation was monitored for three days. All animal experiments were approved by the Ethics Committee of Chongqing Medical University, department Laboratory Animal Management and Use Committee of Chongqing Medical University (IACUC-CQMU) and were conducted in accordance with the regulations for experimental animal management. The acid tolerance test was performed by inoculating the bacteria in acidified LB media. The bacteria were cultured in LB media until the logarithmic growth phase (OD = 0.6), after which 1 μL of the culture was added to 100 μL of fresh acidified LB media (pH = 4.0) and incubated at 37°C for 0, 10, 20, and 30 hours. Samples were taken at different time points and spread on LB plates. After 18 hours, the number of colonies was counted, and the PFU of the bacteria was calculated. Each group was repeated three times.

### Evaluation of *E. coli* DH5α△FliC△tnaA2a and virtual experiment in laboratory teaching as a substitute of *S. flexneri* 2a

A total of 10 teachers, 5 medical laboratory staff from the hospital, and students from three experimental classes were invited to identify *E. coli* DH5α△FliC△tnaA2a and *S. flexneri* 2a bacteria using standard identification methods, including biochemical reaction experiments and serological tests. Specifically, the bacteria were first streaked on MAC and SS plates and suspected pathogenic bacterial colonies were then picked for preliminary identification with Gram staining and puncture inoculation in KIA and MIU. Finally, the suspected bacteria were identified based on biochemical phenomena and serological results, and the results were recorded.To test the effectiveness of using *E. coli* DH5α△FliC△tnaA2a as a substitute for real *S. flexneri* 2a in experimental teaching, students with odd student numbers were required to practice with real *S. flexneri* 2a, while the other half practiced with *E. coli* DH5α△FliC△tnaA2a. Students who could not come to school due to the COVID-19 epidemic were required to practice with a virtual experiment system online. At the end of the semester, all students in the three groups were invited to take a mock examination on the identification of Enterobacteriaceae bacteria. The examination consisted of items such as zoning streak plating, selection of the correct colony, and preliminary identification with Gram staining, culture results in KIA and MIU, and results and discussion, with a maximum of 20 points available for each item. Finally, the scores of the three groups were tallied and analyzed.

### Survey instrument development

In our investigation, we surveyed students from the medical laboratory technique and clinical medicine, as well as their teachers. The survey asked students to provide feedback on their experiences in the microbiology laboratory, including their feelings about laboratory classes, safety concerns, and attitudes toward substitute materials used in class. Surveys were conducted using either the school’s Superstar system or paper questionnaires. This study was approved by the Ethics Committee of Chongqing Medical University.

According to our study proposal, the survey of students and teachers was conducted by the researchers who recruited volunteers during the break of the laboratory classes and obtained their informed verbal consent. No incentives were provided to participate and the data obtained from the simulation test does not involve the privacy of participants, nor does it affect the official exam results. The Ethics Committee of Chongqing Medical University reviewed and approved our proposal and provided us with a supporting document (Reference Number: 2023027).

### Statistical analysis

Data from the surveys and the survival rate of the bacteria in acid were analyzed using Graphpad Prism 8.0. The show as average with standard error of mean. Statistical analysis was performed using Student’s t-test and p values less than 0.05 were considered significantly different and indicated by asterisks in the figures.

## Results

### Teacher and students’ attitudes towards laboratory classes and biosafety

The attitudes of teachers and students towards microbiology laboratory classes were evaluated through questionnaires, including biosafety and the most interesting parts of the class.

**Figure 1:**
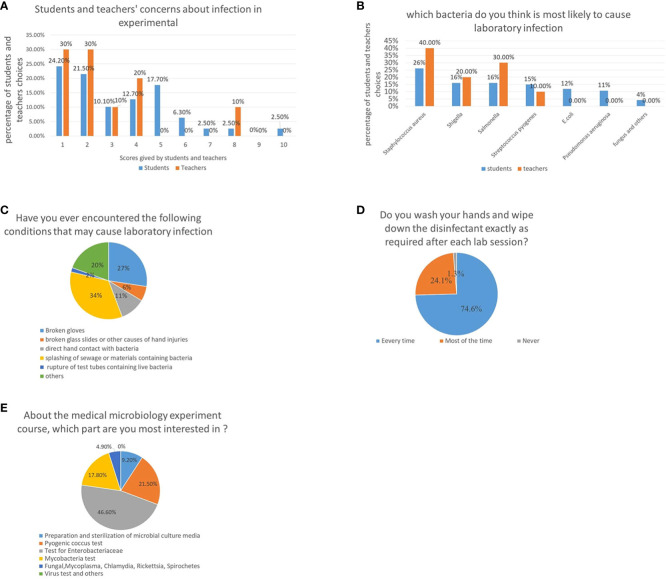
Attitudes of teachers and students toward medical microbiology experiments and potential safety problems. **(A)** Concerns about the laboratory acquired infections in medical microbiology classes, higher scores represent higher levels of worries of students and teachers. **(B)** Microorganisms that teachers and students believe are most likely to cause laboratory infections. **(C)** Unexpected situations encountered by students in a medical microbiology laboratory class. **(D)** Evaluation of students’ awareness on their personal responsibility by investigating whether they washed their hands after class. **(E)** A survey about students’ most interested part of the medical microbiology laboratory course.

Regarding biosafety during class, the students were asked, ‘Do you worry about microbial infections during the experiment?’ The students were told to rate their level of worry from 1 to 10; 1 indicating the least worry. The results show that 24.2, 21.5, 10.1, 12.7, 17.7, and 6.3% of the students chose 1, 2, 3, 4, 5, and 6 points, respectively, while the remaining, 13.8% of the students, chose 7–10 points. Similar to the student’s choice, 30, 30, 10, and 20% of teachers chose one, two, three, and four points, respectively, while 10% chose more than five points, indicating that most students and teachers were moderately worried (**Figure 1A**). When asked about the bacteria which is most likely to cause infection accidents, 26.1% of the students surveyed responded *Staphylococcus aureus*, followed by *Shigella* and *Salmonella*, both with 16.1%. The teachers’ responses were similar to those of the students; 40, 30, 20, and 10% of the teachers chose *S. aureus*, *Salmonella*, *Shigella*, and others, respectively (**Figure 1B**). This showed that both teachers and students had a good understanding of bacterial pathogenicity, and the concerns about biosafety.

To evaluate potential biosafety issues arising during medical microbiology experiments conducted by students, classroom observations and questionnaires on potential biosafety issues were collected. It was observed that student behavior during the class was not appropriate; approximately 1/5^th^ of the classes had incidents requiring help from the teacher, such as a fire caused by an alcohol lamp, and injuries, which may have led to biological contamination. Statistical analysis revealed that majority of the students encountered situations that may have led to infection during the semester-long course. Many students experienced glove tearing (27.4%), hand injuries caused by broken glass (6.2%), direct contact with bacterial cultures (10.6%), being splashed with bacteria-containing materials (34.5%), broken tubes (1.8%), and other hazards (19.5%) (**Figure 1C**).

In addition, students’ awareness about personal responsibility was evaluated by investigating whether they washed their hands after class. Although most students washed their hands after every class (74.6%), some students washed their hands after classes (24.1%) or never (1.3%), indicating that not every student conducted appropriate biosafety procedures (**Figure 1D**).

Owing to the COVID-19 pandemic, some laboratory courses were completed online. Therefore, students’ and teachers’ attitudes towards online virtual experiments was investigated. Most students showed very little interest in purely virtual experiments, instead preferring in-person classes. Approximately 91.1% and 90% of the students and teachers, respectively, opposed purely online virtual experiments.

Finally, a survey to identify the course content that most interested students was conducted. Results showed that Enterobacteriaceae were of the greatest interest, scoring higher than Pyrococcus and non-fermentative bacteria, mainly because the Enterobacteriaceae test procedure is more complex and interesting. It was also the main focus of the course. In comparison, students were least interested in viruses since there were no in-person virology-focused experiments. There was only one virology-based experiment which was completed by viewing videos and pictures taken by the teachers (**Figure 1E**).

In conclusion, most teachers and students prefer in-person medical microbiology laboratory classes, although most of them have encountered potential biosafety problems. Therefore, the use of safe biological materials is necessary. Given the students’ keen interest and, this study focused to simulate *Shigella* by modifying *E. coli* DH5α strain.

### *E. coli* DH5α△tnaA△FliC2a has analogous biochemical characteristics to *S. flexneri 2a*

*E. coli* DH5α strain was adapted to mimic key features of *S. flexneri* 2a by deleting the tnaA and FliC genes and introducing an O-antigen expressing plasmid, resulting in *E. coli* DH5α△tnaA△FliC2a. Additional details are provided in **Supplementary Data 1**.

**Figure 2:**
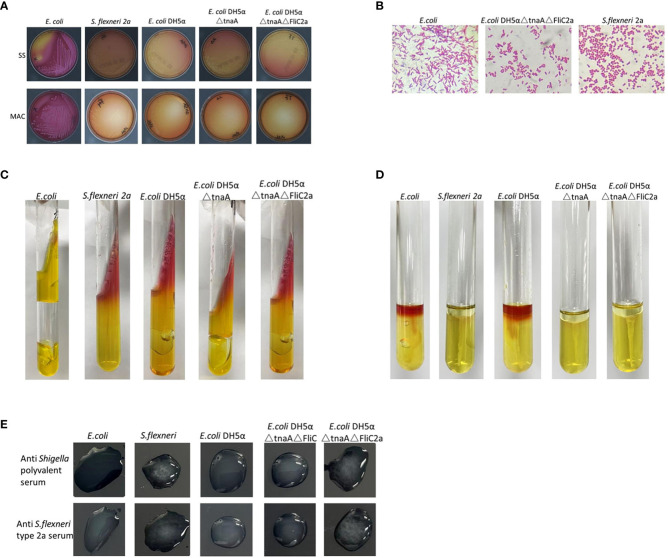
Comparison of biochemical and serological characteristics of bacterium *E. coli* DH5α△tnaA△FliC2a and *S. flexneri* 2a. **(A)**. Colonies of *E. coli*, *S. flexneri* 2a, *E. coli* DH5α, *E. coli* DH5α△tnaA, and *E. coli* DH5α△tnaA△FliC2a on SS (top) and MAC (bottom) agar plates. Only colonies of *E. coli* displayed pink coloration. **(B)** Gram staining images of *E. coli*, *E. coli* DH5α△tnaA△FliC2a and *S. flexneri* 2a. **(C, D)** Inoculation results of *E. coli*, *S. flexneri* 2a, *E. coli* DH5α, *E. coli* DH5α△tnaA and *E. coli* DH5α△tnaA△FliC2a (from left to right) in the KIA and MIU media. **(E)** Results of serological test of *E. coli*, *S. flexneri* 2a, *E. coli* DH5α△tnaA△FliC and *E. coli* DH5α△tnaA△FliC2a with the anti-*Shigella* polyvalent (upper row) and anti *S. flexneri* type 2a serums (lower row).

As expected, *E. coli* DH5α△tnaA△FliC2a had characteristics similar to *S. flexneri* 2a, as shown by Gram staining and biochemical experiments. On MAC and SS agar plates, *E. coli* DH5α derived strains and *S. flexneri* 2a were colorless and translucent, while *E. coli* produced pink colonies (**Figure 2A**). This demonstrated the loss of lactose decomposition ability in *E. coli* DH5α△tnaA△FliC2a and other DH5α-derived strains, which are representative of intestinal pathogenic bacteria, such as *Shigella* and *Salmonella*.

Under a light microscope, *E. coli*, *E. coli* DH5α, *S. flexneri* 2a, and *E. coli* DH5α△tnaA△FliC2a showed identical Gram-negative bacilli scattered in the array. *E. coli* DH5α-derived cells were slightly larger than *Shigella* cells, but shorter than *E. coli*; however, this size difference was not enough to distinguish them (**Figure 2B**).

The characteristics observed in KIA and MIU media were key diagnostic features used to differentiate *Shigella* from other Enterobacteriaceae. Generally, the characteristics of *E. coli* on KIA are AA+-, while those of *Shigella* are KA-/+-. In this part, *E. coli, E. coli* DH5α, *E. coli* DH5α△tnaA, *E. coli* DH5α△tnaA△FliC2a, and *Shigella* in KIA media were tested and compared. The results were similar to those expected, except that compared to *Shigella*, the *E. coli* DH5α-derived strains produced lesser gas, but significantly less as compared with *E. coli*. Generally, only some *Shigella* strains produce gas in KIA. These DH5α-derived strains are not perfect but they closely simulate the characteristics of *Shigella* in KIA (**Figure 2C**). Their characteristics in the MIU media were as anticipated; *E. coli* and *E. coli* DH5α in MIU media showed ++-, those of *E. coli* DH5α△tnaA were +–, and of *E. coli* DH5α△tnaA△FliC2a and *Shigella* were —. This illustrates that *E. coli* DH5α△tnaA△FliC2a can effectively replace *S. flexneri* 2a in MIU experiments (**Figure 2D**).

Further, the serological characteristics of *E. coli*, *E. coli* DH5α, *S. flexneri* 2a, *E. coli* DH5α△tnaA△FliC, and *E. coli* DH5α△tnaA△FliC2a were tested with the *Shigella* serological test kit. Only *S. flexneri* 2a and *E. coli* DH5α△tnaA△FliC2a agglutinated with *Shigella* multivalent serum and *S. flexneri* 2a serum (**Figure 2E**). Additionally, *E. coli* DH5α△tnaA△FliC2a samples were sent to five hospital laboratories for blind testing in order to further confirm its characteristics, where each hospital identified it as *S. flexneri*.

These results indicate that *E. coli* DH5α△tnaA△FliC2a can effectively simulate *S. flexneri* 2a in terms of its key biological characteristics.

### *E. coli* DH5α△tnaA△FliC2a has lower viability than *E. coli* and lower toxicity than *S. flexneri* 2a

Although *E. coli* DH5α△tnaA△FliC2a effectively simulated *S. flexneri* 2a, it was necessary to confirm its safety. Given *Shigella*’s intolerance to acid and pathogenicity to guinea pigs, acid resistance and guinea pig eye toxicity testing were carried out to determine the resistance and toxicity of *E. coli* DH5α△tnaA△FliC2a, respectively.

**Figure 3:**
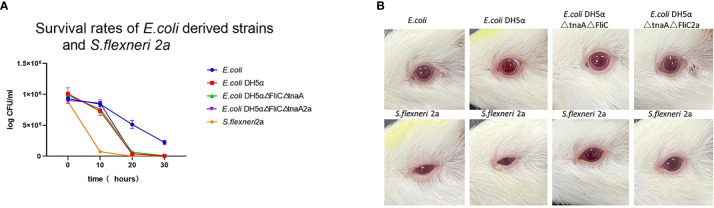
*E. coli* DH5α△tnaA△FliC2a has lower viability than wild type *E. coli* and lower toxicity than *S. flexneri* 2a. **(A)** In the acid tolerance test, 1x10^6^ PFU/mL of *E. coli* (Blue line plotted with circles), *E. coli* DH5α (red line plotted with squares) and its derivatives (green line plotted with upright triangles and purple line plotted with inverted triangles, separately) were cultured in acidified LB media and the plaques were static at 0, 10, 20 and 30 hours. **(B)** In the guinea pig eye toxicity test, *E. coli*, *E. coli* DH5α, *E. coli* DH5α△tnaA△FliC and *E. coli* DH5α△tnaA△FliC2a inoculated groups (upper row) showed no signs of inflammation, whereas all the 4 animals inoculated with *S. flexneri* 2a (lower row) showed inflammatory symptoms such as increased secretion, redness, and inability to open the eyelids.

*E. coli*, *E. coli* DH5α, *E. coli* DH5α△FliC△tnaA, *E. coli* DH5α△FliC△tnaA2a, and *S. flexneri* 2a were placed in acidic LB media (pH 4.0) to monitor their survival rate for 30 h; this showed that bacterial colonies formed, decreased with increased incubation time. *E. coli* had the highest survival rate after 30 h (23.7%), followed by *E. coli* DH5α (0.42%), *E. coli* DH5α△FliC△tnaA (0.82%), and *E. coli* DH5α△FliC△tnaA2a (0.25%). Almost all *S. flexneri* 2a were killed after 30 h (**Figure 3A**). This indicated that DH5α-derived strains had intermediate resistance to acid, that is between *S. flexneri* 2a and *E. coli*. Since *E. coli* is considered safe when used in student experiments, *E. coli* DH5α and its derived strains should be safer.

To further confirm the safety of *E. coli* DH5α△FliC△tnaA2a, guinea pig eye toxicity tests were carried out. Each strain was inoculated into the eyes of guinea pigs. After 72 h, all four guinea pigs inoculated with *S. flexneri* 2a had inflammatory symptoms, such as redness, blurred eyes, and massive secretions in both eyes, while guinea pigs inoculated with the other strains showed no inflammatory symptoms (**Figure 3B**). This shows that *E. coli* DH5α△tnaA△FliC2a, similar to *E. coli* and *E. coli* DH5, had lesser bio-hazard than *S. flexneri* 2a.

### *E. coli* DH5α△tnaA△FliC2a is equally effective as *Shigella* in medical microbiology laboratory classes

**Figure 4:**
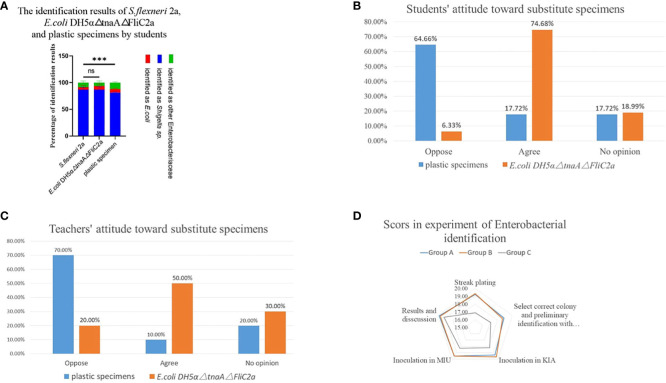
*E. coli* DH5α△tnaA△FliC2a has similar teaching effect with *S. flexneri* 2a in medical microbiology laboratory class. **(A)** The identification results of *S. flexneri* 2a, *E. coli* DH5α△tnaA△FliC2a and plastic specimens by students. **(B, C)** Students’ and teachers’ attitudes of replacing *S. flexneri* 2a with plastic specimens and *E. coli* DH5α△tnaA△FliC2a in medical microbiology laboratory class. **(D)** Students practicing with *S. flexneri* 2a (Group A) and *E. coli* DH5α△tnaA△FliC2a (Group B) outperformed those only participating in simulated experiments (Group C) in terms of their scores on zoning streak, colony selection, KIA and MIU inoculation and identification result analysis. Data above were shown as mean ± SEM of n = 3 replicates, student’s t-test: ***p < 0.001; NS, not significant.

Since *E. coli* DH5α△tnaA△FliC2a had greater biosafety and analogous biochemical and biological characteristics as *S. flexneri* 2a, its effectiveness in teaching in comparison to *S. flexneri* 2a and plastic specimens was evaluated. Experimental reports submitted by students in three independent groups showed that most students accurately identified *S. flexneri* 2a (86.84%), *E. coli* DH5α△tnaA△FliC2a (86.99%), and plastic specimens (81.33%) as *Shigella* sp., and only a few incorrectly identified the specimens as *E. coli* (4.66%, 6.44%, and 6.54%, respectively) or other Enterobacteriaceae (85.0%, 65.70%, and 12.15%, respectively) (**Figure 4A**).

Further, the attitudes of teachers and students towards *E. coli* DH5α△tnaA△FliC2a and plastic specimens was investigated. According to the survey of students’ attitudes towards simulation materials, 17.72% of the students believed that plastic specimens should be used instead of real bacteria, 17.72% had no opinion, while 64.66% opposed the use of plastic specimens. The major objections were that the plastic specimens looked too fake and that it was impossible to carry out certain operations, such as streaking, inoculation and bacterial culture. In contrast, 74.68% of the students agreed to substitution of *Shigella* with *E. coli* DH5α△tnaA△FliC2a, while 18.19% had no opinion, and only 6.33% opposed the substitution (**Figure 4B**). Subsequently, a survey on teachers’ attitudes was conducted, which showed that 10% of the teachers supported plastic specimen use, 20% had no opinion while 70% opposed the idea. For *E. coli* DH5α△tnaA△FliC2a, half of the teachers supported its use, 30% had no opinion, and 20% opposed its use (**Figure 4C**). Teachers had reasons similar to students, including that plastic specimens can only be observed, but some who supported the use of plastic specimens also cited safety considerations.

Students’ scores also reflected the objective effect of *E. coli* DH5α△tnaA△FliC2a in laboratory teaching. Students were divided into three groups to practice Enterobacteriaceae identification. Groups A and B practiced with *E. coli* DH5α△tnaA△FliC2a and *S. flexneri* 2a, respectively, while group C was composed of students who could not return to school because of the COVID-19 pandemic and therefore practiced through online virtual experiments. A simulation test was conducted and the scores were analyzed. The test involved various operations, including Gram staining, single colony isolation (at least five single colonies), selecting suspicious colonies for Gram staining and culture, inoculation, cultivation, interpreting KIA and MIU media results, and explaining the experimental results to teachers, each item was assigned a score out of 20. As expected, the average scores of groups A and B for each criterion were similar but significantly higher than that of group C.

Students in groups A and B scored an average of 19.22 and 19.32 points, respectively, in zoning streak plating, while the students in group C only scored 16.9 points. For selecting suspicious colonies and subsequent preliminary identification after Gram staining, groups A and B scored 18.87 and 18.68 points on average, respectively, while group C scored 17.10 points. In terms of interpretation and results, the scores were relatively close between the three groups; the average scores of group C were 18.20 and 18.30, lower than those of groups A (19.25 and 19.52 points) and B (19.63 and 19.53 points). The average scores of groups A, B, and C were the closest for interpretation and discussion of the results at 19.83, 19.68, and 19.20 points, respectively (**Figures 4D**, **S2**). In terms of overall grades, students in groups A and B achieved average scores of 96.78% and 96.84%, respectively, while group C obtained an average score of 89.70% (**Figure S2**).

## Discussion

Medical microbiology laboratory course involves potential biosafety risks due to the interaction between inexperienced undergraduate students and live pathogenic microorganisms. Lack of strict training and an imbalance in the teacher to student ratio increases these risks. The development of CRISPR/Cas9 technology for gene manipulation has made it easier than ever to edit the genomes of microbes and are already used in vaccine production and bioengineering industry (Jiang et al., 2015; Yan et al., 2017; Zhang et al., 2019; Hashemi, 2020; Wang et al., 2020; Yan et al., 2020). This progress also allows new ideas to address the biosafety issues in medical microbiology laboratory teaching.

The CRISPR/Cas9 gene editing technique was applied to *E.coli* DH5α, a safe engineering bacteria, to mimic highly pathogenic *Shigella* (Jang et al., 2017; Besser, 2018; Lampel et al., 2018; Wang et al., 2021; Kotloff, 2022). The modified *E. coli* DH5α△tnaA△FliC2a strain can replicate almost all of the key characteristics of *S. flexneri* 2a. The only drawback being the gas production in KIA media, which is much less than that of *E. coli*, yet similar to some *Salmonella* strains. Given that some *Shigella* strains also produce small amounts of gas in KIA media, such a small quantity is not enough to affect identification using the Key Table for Identification of Enterobacteriaceae. This issue may be resolved in future by modifying the genes related to gas production.

Further, the effect of *E. coli* DH5α△tnaA△FliC2a, plastic specimens and online virtual experiments on teaching was assessed. In contrast to previous research showing that students generally accept plastic models (Jia et al., 2022), this investigation revealed that most students opposed their use. This difference could be attributed to the fact that participants of the former research majored in nursing, medical imaging, and other fields that only necessitate observation of microorganisms, as opposed to clinical medicine and medical laboratory technology, wherein students must culture and isolate microorganisms, as well as independently authenticate their serology. Majority of the teachers and students believed that the plastic samples simulating the results of each step did not adequately replace the actual bacteria b whereas, *E. coli* DH5α△tnaA△FliC2a strain possibly could.

In case of virtual experiments, most teachers and students were willing to accept them; however, given an option most study participants prefer in person laboratory classes. The simulation tests also revealed that students who practiced using virtual experiments fared worse in the assessment than those using *E. coli* DH5α△tnaA△FliC2a. Nevertheless, this method should only serve as an auxiliary exercise prior to a laboratory class, or as an auxiliary teaching method in emergent circumstances, such as epidemics, and should not replace actual experiments. These findings are in accordance with previous research (Brockman et al., 2020; Joji et al., 2022).

**Figure 5:**
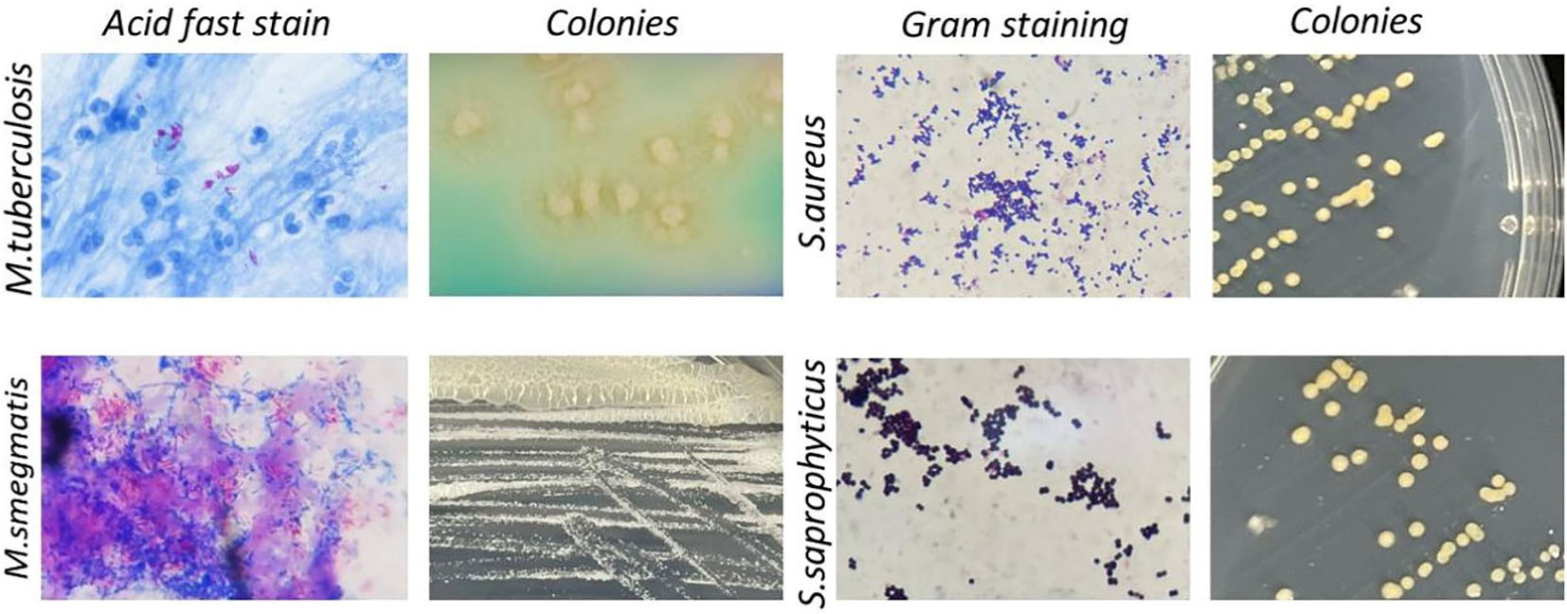
Summary of common bacteria strains and alternatives in medical microbiological laboratory teaching. Left, Acid-fast staining and colony of *M. tuberculosis* (upper row) and its safer alternative *M. smegmatis* (lower row). Right, Gram staining and colony of *S. aureus* (upper row) and its safer alternative *S. epidermidis* (lower row).

In addition to *Shigella*, medical microbiology laboratory classes also involve other highly pathogenic microorganisms. Based on the difficulties with biosafety, cultivation, regulation, experimental conditions, and legal requirements, the use of alternative strains is common, but existing alternatives have notable shortcomings. For example, due to low pathogenicity and easy cultivation, *Mycobacterium segments* have replaced *M. tuberculosis* in medical microbiology experiments for acid-fast staining (**Figure 5** left first); however, these strains differ significantly in their colony morphology (**Figure 5** left second). While *Staphylococcus saprophyticus* was used to replace *S. aureus* for teaching Gram staining (**Figure 5** right second), the key plasma coagulase assay during *Staphylococcus* identification and colony observation cannot be completely replaced (**Figure 5** right first). The common pathogenic microorganisms involved in medical microbiology laboratory classes and the potential application of the strategy described herein, have been summarized in **Table 1**. This approach could be further applied to these commonly used pathogenic microorganisms to reduce the biosafety risk. The experimental bacterial strain bank with typical biological characteristics that do not present any biosafety risks could be constructed in future.

**Table 1:** Pathogenic microorganisms involved in medical microbiology laboratory class and the potential application objects and methods of our strategy.

Chapters	Experiment content	Microorganisms	Biosecurity level (BSL)	Regular alternatives	Our strategy
Enterobacteriaceae bacteria	bacterial cultivation, Gram staining, biochemical experiments and serological experiments	*E.coli*	1	Needless	Needless
		*Shigella flexneri 2a*	2	None	*E. coli* DH5α△FliC△tnaA2a(Finished)
		*Salmonella typhi*	2	None	Modifying *E. coli* DH5α or attenuated *S.typhimurium*
pathogenic cocci	bacterial cultivation, Gram staining and serological experiments	*S. aureus*	2	a regular alternative method for Gram stain is to use the S.saprophyticus	attenuated *S.aureus*
		*S.epidermidis*	1	None	attenuated *S.epidermidis*
		*S.saprophyticus*	1	Needless	Needless
Streptococcus	bacterial cultivation, Gram staining and serological experiments	α-Hemolytic streptococcus	2	None/plastic models	None
		β-Hemolytic streptococcus	2	None/plastic models	None
		γ-Streptococcus	1	Needless	Needless
		*S.pneumoniae*	2	None	None
non-fermenting bacteria	bacterial cultivation, Gram staining, biochemical experiments	*Pseudomonas aeruginosa*	2	None	None
		*Acinetobacter baumannii*	2	None	None
		*Stenotrophomonas maltophilia*	1	Needless	Needless
Anaerobes	cultivation and observation of oral anaerobic bacteria	oral anaerobic bacteria	–	Needless	Needless
*Mycobacterium*	acid-fast staining and colony characteristic observation	*M. tuberculosis*	3	a regular alternative method for acid-fast staining is to use the *Mycolicibacterium smegmati*s instead	Bacillus Calmette Guerin
Spirochetes	morphology observation	*Borrelia vincenti*	–	Needless	Needless
Fungi	observation of fungal colony morphology and spores	Pichia pastoris and other fungi separated from air	–	Needless	Needless
Virology	Character of virus, cell pathogenic effect	no suitable virus	2 to 4	pictures/teaching videos	Modifying insect baculovirus AcNPV

## Data availability statement

The original contributions presented in the study are included in the article/**Supplementary Material**. Further inquiries can be directed to the corresponding author.

## Ethics statement

The studies involving humans were approved by Ethics Committee of Chongqing Medical University. The studies were conducted in accordance with the local legislation and institutional requirements. The participants provided their written informed consent to participate in this study. The animal studies were approved by Ethics Committee of Chongqing Medical University, department Laboratory Animal Management and Use Committee of Chongqing Medical University (IACUC-CQMU). The studies were conducted in accordance with the local legislation and institutional requirements. Written informed consent was obtained from the owners for the participation of their animals in this study.

## Author contributions

GZ: Investigation, Writing – original draft. JL: Writing – original draft, Data curation, Methodology. YHe: Conceptualization, Investigation, Writing – review & editing. YD: Formal Analysis, Writing – original draft. LX: Writing – original draft, Methodology, Software. TC: Writing – original draft, Funding acquisition, Validation. YG: Data curation, Methodology, Writing – review & editing. HF: Resources, Validation, Writing – original draft. AL: Writing – original draft, Formal Analysis, Software. YT: Writing – original draft, Funding acquisition, Methodology. YHu: Writing – original draft, Resources, Validation. CY: Data curation, Writing – review & editing. ML: Methodology, Project administration, Writing – original draft. XD: Methodology, Data curation, Writing – review & editing. JW: Writing – review & editing, Resources, Software. NL: Conceptualization, Funding acquisition, Supervision, Writing – original draft.
