# Supplementary material for: Modifying Escherichia coli to mimic Shigella for medical microbiology laboratory teaching: a new strategy to improve biosafety in class
Source: Front Cell Infect Microbiol. 2023 Sep 13;13:1257361. doi: 10.3389/fcimb.2023.1257361 (PMC10533986; doi:10.3389/fcimb.2023.1257361)
Supplement: Supplementary file 1 [file DataSheet_1.docx]

**Supplementary data**

**Table S1. Primers used in this research.**

| Name | Sequence |
| --- | --- |
| tnaA-updonor-F | gccttttgcaaaggtcatctctcg |
| tnaA-updonor-R | ctctgtagtattaagcggaacggttcagggagatg |
| tnaA-downdonor-F | gttccgcttaatactacagagtggctataaggatg |
| tnaA-downdonor-R | cctccaccaattactgtacctgcta |
| FliC-updonor-F | GCGGGAATAAGGGGCAGAGA |
| FliC -updonor-R | CGATTAACCCTGCAGCAGAGAGTATTAATGACTTGTGCCATGATTCG |
| FliC -downdonor-F | TCTCTGCTGCAGGGTTAATCG |
| FliC -downdonor-R | GGGGTTATTTGGGGGTTACAGG |
| TAR-tnaA-F | actagtgtgaagcagaatacaaagacgttttagagctagaaatagcaagttaaa |
| TAR-tnaA-R | ctaaaacgtctttgtattctgcttcacactagtattatacctaggactgagctag |
| TAR-FliC-F | actagtTCACCGGTGGTGATAACGATgttttagagctagaaatagcaagttaaa |
| TAR-FliC-R | taaaacATCGTTATCACCACCGGTGAactagtattatacctaggactgagctag |
| pTarget-seq-F | gcagcgagtcagtgagcgag |
| T7-ter-F(ln) | tgaggtaccggACTAGTGTTAACTAGCATAACCCCTTG |
| pOcus2-R(ln) | TCAGATCTTTCTGTCATAGTCCATTTTGGGTCTAGAATCCCCTTCCTCGCTCACTG |
| bgt-F(ln) | ACTATGACAGAAAGATCTGATATTTTTCCTCGCAAAAATGAAAATATCTCTTGTCGTTC |
| grtII-R(ln) | CAGATTTTCATTAGGATCCCTAAATATTAAATGGAAGCCACCCT |
| rfbB-F | TTAGGGATCCTAATGAAAATCTGACCGGATGTAACGGTTG |
| rfbC-R | TTAACACTAGTCCGGTACCTCACTCGAGCGAGAAATCCTAGCGA |

**Methods:**

## Generation of tnaA and FliC gene deficient *E. coli* DH5α△TnaA△FliC

The genome of *E. coli* strain DH5α was modified using CRISPR/Cas9 technology to eliminate the tnaA and FliC genes. The names and sequences of primers used are listed in Table S1. First, the upstream and downstream fragments next to the tnaA ORF were separately amplified using the primers tnaA-updonor-F/tnaA-updonor-R and tnaA-updonor-F/tnaA-downdonor-R. The two fragments were then mixed together as a template and amplified with the primers tnaA-updonor-F/tnaA-downdonor-R to generate the tnaA-donor fragment. The fragment was recovered by gel cutting after electrophoresis detection.
The plasmid pTarget-tnaA, which contains the sgDNA sequence targeting the tnaA gene, was constructed using the one-step annealing method. Specifically, the plasmid pTarget (Addgene, Plasmid #62226) was amplified with the primers TAR-tnaA-F/TAR-tnaA-R. The PCR product was then digested with DpnI and transformed into *E. coli* DH5α competent cells. The transformed bacteria were plated on spectinomycin (Spe) LB plates and incubated at 37℃ overnight. After 18 hours, single colonies were picked, and their sequences were confirmed by Sanger sequencing using the primer pTarget-seq-F.
The L-arabinose-induced *E. coli* DH5α electro-competent cells carrying the pCas9 (Cas9 expression) plasmid were purchased from KBL Ltd. (Chongqing, China). A 200 ng mixture of pTarget-tnaA plasmid and tnaA-donor fragments was electro-transformed into the competent cells. The bacterial fluid was then spread and cultured on LB plates containing kanamycin (Kan) and Spe at 30℃. The tnaA gene knockout colonies were identified by PCR amplification with the primers tnaA-updonor-F/tnaA-downdonor-R. The plasmids pTarget-tnaA and pCas9 were eliminated by subculturing the bacteria in LB without antibiotics at 42℃ for 48 hours and further identified by an antibiotic-sensitive test. The modified bacteria were named *E. coli* DH5αΔtnaA.
To generate the tnaA and FliC gene dual knockout in E. coli DH5α, we constructed the plasmid pTarget-FliC targeting the FliC gene and the donor fragment FliC-donor using a similar method mentioned above. Specifically, the plasmid pTarget was amplified with the primers TAR-FliC-F/R, and the PCR products were digested with DpnI and transformed into *E. coli* DH5α to generate pTarget-FliC. The donor fragment FliC-donor was generated by amplifying the upstream and downstream fragments of the FliC ORF using primers FliC-updonor-F/R, FliC-downdonor-F/R, and annealing the products by overlapping PCR. On the other hand, the pCas9 plasmid was transformed into E. coli DH5αΔtnaA, and the transformed bacteria were induced by L-arabinose and prepared as electro-competent cells. The competent cells were then electro-transformed with 200 ng of pTarget-FliC plasmid and donor fragment FliC-donor and plated on Kan and Spe plates. The TnaA and FliC dual knockout bacteria were identified by PCR using primers FliC-updonor-F and FliC.

**Results:**

**Identification of *E.coli* DH5α△tnaA△FliC2a**

To simulate the key biochemical reaction characteristics of Shigella, the tryptophanase, flagella genes, and lactose metabolism-related genes of E. coli need to be knocked out, and the Shigella antigenic determinant gene needs to be introduced. Since E. coli DH5α is originally lactose operon-deficient, according to the analysis results in KEGG, only the tryptophanase (tnaA) and flagella (FliC) genes should be knocked out (Fig. S1A and B). First, the tnaA gene was knocked out using the CRISPR/Cas9 system and identified using PCR. The result showed that compared with the wild-type E. coli DH5α (Figure 1C, lane 11), the tnaA knocked-out strain has smaller bands (Fig. S1C, lane 1). Thus, we were fortunate to obtain the only tnaA knock-out clone, and the other 10 clones failed (Fig. S1C, band 2-10). After plasmid elimination, the bacteria were named *E. coli* DH5α△tnaA. The tnaA and FliC gene dual knocked-out strain was constructed based on *E. coli* DH5α△tnaA and identified by PCR. The FliC gene knocked-out strains have smaller bands than that of the wild-type (Figure 1D, lane 1, 6-10). Then all the plasmids were eliminated, and the bacteria were named E. coli DH5α△tnaA△FliC. The S. flexneri 2a O antigen expression plasmids were transformed into the E. coli DH5α△tnaA△FliC, and the S. flexneri 2a antigen expression was identified by the Shigella serological test kit, and the transformed bacteria were named E. coli DH5α△tnaA△FliC2a. As expected, only E. coli DH5α△tnaA△FliC2a and S. flexneri 2a agglutinated with the anti-Shigella multivalent mixed serum and anti-S. flexneri 2a monovalent serum, while *E. coli* and *E. coli* DH5α could not (the results are shown in the next section).

**Fig. S1**


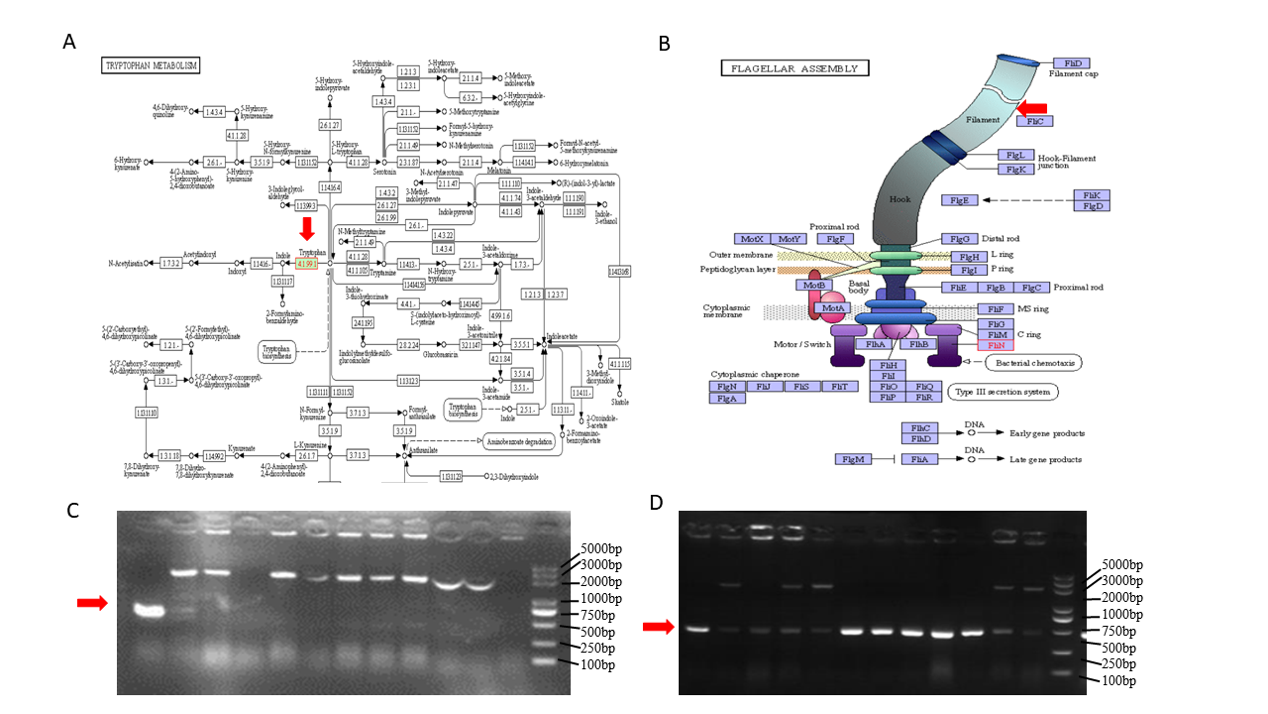


**Fig. S2**


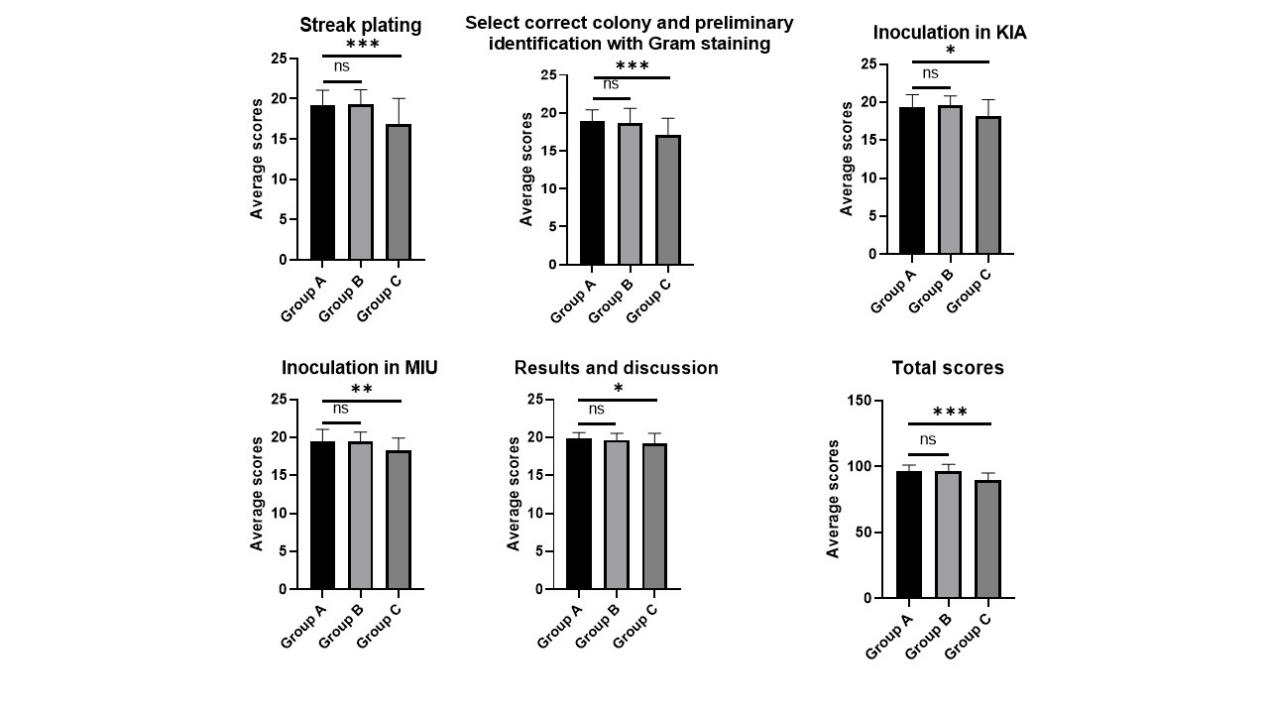


**Fig. S1.** Identification of *E.coli* DH5α△tnaA△FliC2a. (A) Analysis of tryptophan metabolism in *E. coli* K12 MG1655 using the KEGG database revealed that tryptophanase (depicted as the red arrow) encoded by the tnaA gene is the only enzyme which catalyzes the conversion of tryptophan to indole in this pathway. (B) Analysis of flagellar assembly in *E. coli* K12 MG1655 using the KEGG database revealed that Filament (depicted as the red arrow) encoded by FliC gene is the main protein that constitutes flagella. (C) PCR identification of tnaA-knocked out *E.coli* DH5α△tnaA. Lane 1 (from left to right, indicated by the red arrow) demonstrates an apparent smaller amplification product in tnaA-deficient bacteria as compared to that in wild-type bacteria (Lanes 2–12), while Lane 13 represents the DNA ladder. (D) PCR identification of TnaA and FliC-knocked out *E.coli* DH5α△tnaA△FliC. Lane 1, 3, 6 to 10 (from left to right, indicated by the red arrow) demonstrates an apparent single smaller amplification product in FliC-deficient bacteria as compared to that in wild-type bacteria or non-monoclonal bacteria (Lanes 2, 4, 5, 11 and 12), while Lane 13 represents the DNA ladder.

**Fig. S2.** Students practicing with *S.flexneri* 2a (Group A) and *E.coli* DH5α△tnaA△FliC2a (Group B) outperformed those only participating in simulated experiments (Group C) in terms of their scores (20 point for each item) on zoning streak, colony selection, KIA and MIU inoculation and results and discussion, and the total scores in average. All data above were shown as mean ± SEM of n = 3 replicates, student’s t-test: *p < 0.1; **p < 0.01; ***p < 0.001; NS, not significant.
